# Popliteal Artery Occlusion Due to Femur Exostosis in a Patient With Rheumatoid Arthritis: A Rare Cause of Chronic Limb-Threatening Ischemia

**DOI:** 10.7759/cureus.69926

**Published:** 2024-09-22

**Authors:** Musaad AlHamzah, Ibrahim Alshaygy, Tariq Alanezi, Mohamed K Bedaiwi

**Affiliations:** 1 Department of Surgery, King Saud University, Riyadh, SAU; 2 Department of Orthopedics, King Saud University, Riyadh, SAU; 3 Department of Medicine, King Saud University, Riyadh, SAU

**Keywords:** bony exostosis, critical limb threatening ischemia, rheumatoid arthriitis, vascular compression, vascular occlusion

## Abstract

Bony exostoses, or osteochondromas, are benign bone tumors that usually develop at growth plates during the growth period. Large exostosis can compress nearby vascular structures, causing occlusion, perforation, or pseudoaneurysm. We report a case of a middle-aged woman with rheumatoid arthritis (RA) and atherosclerotic risk factors who had an unusual presentation of chronic limb-threatening ischemia (CLTI) of the left leg combined with a flare-up of RA. She was found to have an occluded left popliteal artery due to a large femur exostosis. An uncomplicated left femoral-to-popliteal bypass was performed using a reversed great saphenous vein without excision of exostosis. Her ischemic symptoms have been resolved during a 30-month follow-up period. A high index of suspicion is required in such a case to reach the correct diagnosis and prevent catastrophic limb loss.

## Introduction

Exostoses, also known as osteochondromas, are common, benign bone tumors that appear during the bone’s growth and extend outward from an ectopic growth cartilage that develops and matures through the normal endochondral ossification process. Therefore, they are often diagnosed in late childhood or early adulthood. They are solitary in most cases or could be part of multiple hereditary exostoses [1,2]. The mechanism by which exostoses develop is not well understood, but it possibly involves subperiosteal cartilage development due to injury, radiation, surgical interventions, or other triggers. This evolves with time by ossification [3-6]. Vascular complications of exostosis include pseudoaneurysm from repetitive injury to the arterial wall, perforation and bleeding from a direct injury by a sharp edge, and thrombosis secondary to compression and arterial wall injury [2]. We present the case of a middle-aged woman who presented with chronic limb-threatening ischemia due to left popliteal artery occlusion caused by compression by a femur exostosis.

## Case presentation

The patient is a 59-year-old woman with a past medical history of rheumatoid arthritis (RA), diabetes mellitus, hypertension, bilateral cataracts, and a hearing deficit requiring hearing aids. She presented to the emergency department with worsening bilateral lower limb pain and edema for the past seven days. She had multiple episodes of this complaint for the past six months, where her left foot was more swollen than the right. She had been to an outside emergency department a few times and was diagnosed with cellulitis, possible osteomyelitis, and rheumatoid flare-up. Antibiotics were unhelpful, and imaging of the foot ruled out an infective process. She was sent to the emergency department from an outside vascular surgery clinic due to the absence of bilateral pedal pulses. In addition, she had swollen and painful joints in her hands for a few days. She had no rest pain but mentioned bilateral knee pain at night and during ambulation. On further questioning, she mentioned that she gets left leg claudication after walking less than 50 m in the past six months and that she was otherwise functionally independent. No family history of early major cardiovascular events or RA were reported, and she had no surgical history of the lower limbs.

**Figure 1:**
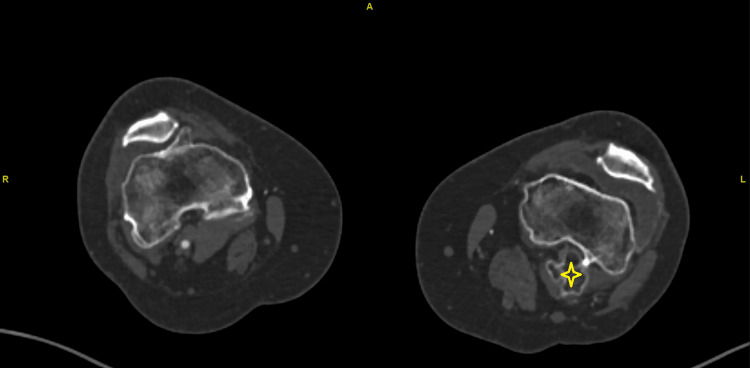
CT slice showing a large left femur exostosis. CT: computed tomography.

On examination, the patient appeared to be in pain in her bed but was hemodynamically stable. The left foot had mild pitting edema with blue discoloration and two pinpoint ulcers at the first webspace. The right foot and both hands were unremarkable. Bilateral radial, brachial, femoral, and popliteal pulses were palpable, along with right pedal biphasic Doppler signals. Left pedal signals were dampened monophasic. Admission for workup and management was offered but declined. She was given an urgent follow-up at the non-invasive vascular laboratory. The Ankle-Brachial Index (ABI) was 0.87 on the right leg and 0.41 on the left leg. A Duplex scan showed an occluded left popliteal artery with reconstitution of the below-knee segment and a mild tibial disease. She had no venous thrombosis. A computed tomography (CT) angiography of the lower limbs confirmed the findings and showed a large exostosis of the posterior aspect of the distal femur measuring 4x3x5 cm (Figure 1).

**Figure 2:**
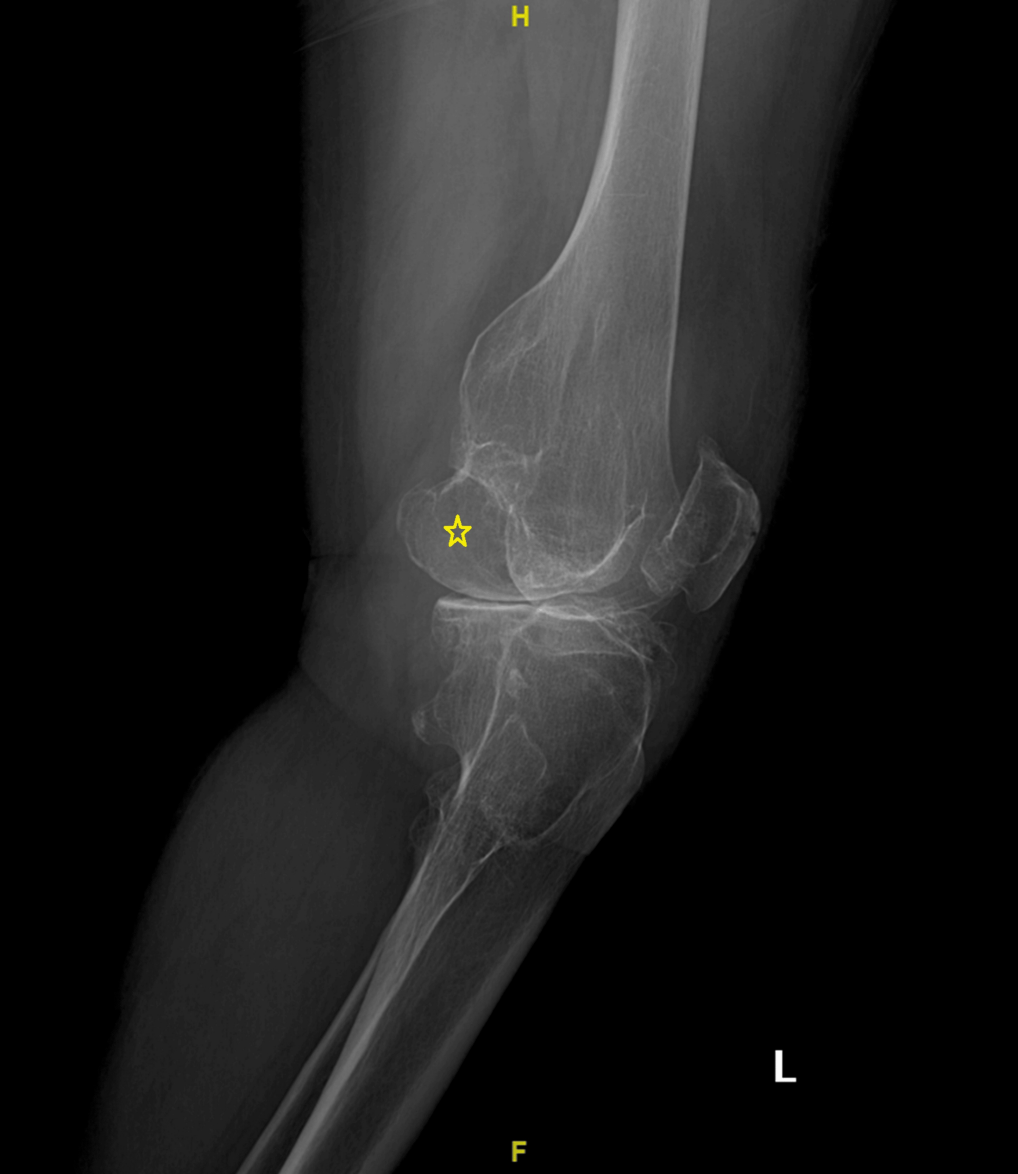
X-ray of the left knee in December 2015, showing the large exostosis.

The popliteal artery reconstitutes just past the compression point by the bone. Furthermore, her white blood cell (WBC) count was above 20,000x10^9^/L, and her platelets count was above 500x10^9^/L for the past two years. She was admitted for urgent evaluation and management and started on a heparin infusion. Chart review showed that she was offered an excision of her left knee exostosis a few years ago but declined the surgery (Figure 2). No other exostoses were identified on skeletal X-rays. 

An orthopedic consultation was made during this presentation, and the decision was made to proceed with an urgent revascularization only due to the higher morbidity of the excision procedure during an active rheumatoid flare-up and the extent of the popliteal artery occlusion causing limb-threatening ischemia.

Rheumatological and hematological consultations were also done, and she was advised to hold surgery for a few days to adjust her medications. She then underwent left femoral-to-popliteal bypass surgery using a reversed left great saphenous vein graft that was tunneled in the subcutaneous plane to avoid the exostosis. The procedure was uncomplicated, and the left foot pain and discoloration were relieved. However, she continued to have leukocytosis, thrombocytosis, and a Janus kinase-2 (JAK2+) mutation. She was diagnosed with polycythemia rubra vera following a bone marrow biopsy and was discharged home on hydroxyurea. During her three-month follow-up visit, she reported no left foot or leg pain, discoloration, or swelling. Her left foot was pink with a palpable left femoral-to-popliteal graft pulse and multiphasic pedal signals. ABI was 0.89 on the right and 0.88 on the left, and the graft surveillance duplex showed a patent graft. Those findings remained stable up until her latest follow-up visit 30 months after surgery, and she will continue to have annual follow-up visits scheduled for graft surveillance.

## Discussion

Vascular complications of bony exostosis are uncommon and include perforation, pseudoaneurysm, and thrombosis, all resulting from compression, repetitive injury, or direct perforation by exostosis [2,3]. As exostoses are often found incidentally, the vascular complications often have an acute onset and are managed urgently [1-3]. Our patient had an uncommon presentation due to her rheumatological disease that was masking her ischemic features and the lack of classical progressive claudication symptoms. However, careful clinical evaluation by a vascular specialist uncovered a short distance claudication symptoms, along with features of limb-threatening ischemia. Furthermore, the exostosis of her distal femur was seen on knee X-rays years before this presentation when the patient's RA was not active, but the patient opted for non-operative therapy. She continued to attend her follow-up visits with her rheumatologist but not the orthopedic surgeon.

Many reported cases of exostosis causing vascular complications suggest that the optimal approach for managing these conditions involves a combination of vascular and orthopedic surgeries, i.e. excision of exostosis and vascular reconstruction by direct repair, end-to-end anastomosis, or a surgical bypass. A thromboembolectomy or thrombolysis, if needed, was also utilized [2,7-14]. In our case, we decided against the excision of exostosis due to active rheumatoid disease, severe ischemic symptoms, and suspicion of a prothrombotic disease, which was confirmed as polycythemia rubra vera. This required tunneling the graft in the subcutaneous plane to avoid any potential compression by the exostosis. Follow-up by rheumatology and vascular surgery until 30 months to date has shown a stable exostosis and a viable limb.

## Conclusions

An exostosis of the distal femur causing a popliteal artery occlusion is a difficult etiology for a RA patient who presents with a recurrent flare-up of the disease, mimicking symptoms of CLTI with several underlying and long-term comorbidities. With an increased risk of thrombosis due to diabetes, hypertension, dyslipidemia, and polycythemia rubra vera, revascularization should be the primary focus of intervention. Excision of exostosis may not be acutely necessary, but regular follow-up must be undertaken to monitor its growth and prevent further complications. Overall, a proper and prompt diagnosis is crucial to avoid severe vascular complications and the subsequent risk of amputation due to bony anomalies.
